# The AGXX® Antimicrobial Coating Causes a Thiol-Specific Oxidative Stress Response and Protein *S*-bacillithiolation in *Staphylococcus aureus*

**DOI:** 10.3389/fmicb.2018.03037

**Published:** 2018-12-11

**Authors:** Vu Van Loi, Tobias Busche, Thalia Preuß, Jörn Kalinowski, Jörg Bernhardt, Haike Antelmann

**Affiliations:** ^1^Institute for Biology-Microbiology, Freie Universität Berlin, Berlin, Germany; ^2^Center for Biotechnology, Bielefeld University, Bielefeld, Germany; ^3^Institute for Microbiology, University of Greifswald, Greifswald, Germany

**Keywords:** *Staphylococcus aureus*, AGXX®, transcriptome, bacillithiol, protein *S*-bacillithiolation

## Abstract

Multidrug-resistant pathogens, such as methicillin-resistant *Staphylococcus aureus* (MRSA) pose an increasing health burden and demand alternative antimicrobials to treat bacterial infections. The surface coating AGXX® is a novel broad-spectrum antimicrobial composed of two transition metals, silver and ruthenium that can be electroplated on various surfaces, such as medical devices and implants. AGXX® has been shown to kill nosocomial and waterborne pathogens by production of reactive oxygen species (ROS), but the effect of AGXX® on the bacterial redox balance has not been demonstrated. Since treatment options for MRSA infections are limited, ROS-producing agents are attractive alternatives to combat multi-resistant strains. In this work, we used RNA-seq transcriptomics, redox biosensor measurements and phenotype analyses to study the mode of action of AGXX® microparticles in *S. aureus* USA300. Using growth and survival assays, the growth-inhibitory amount of AGXX® microparticles was determined as 5 μg/ml. In the RNA-seq transcriptome, AGXX® caused a strong thiol-specific oxidative stress response and protein damage as revealed by the induction of the PerR, HypR, QsrR, MhqR, CstR, CtsR, and HrcA regulons. The derepression of the Fur, Zur, and CsoR regulons indicates that AGXX® also interferes with the metal ion homeostasis inducing Fe^2+^- and Zn^2+^-starvation responses as well as export systems for toxic Ag^+^ ions. The induction of the SigB and GraRS regulons reveals also cell wall and general stress responses. AGXX® stress was further shown to cause protein *S*-bacillithiolation, protein aggregation and an oxidative shift in the bacillithiol (BSH) redox potential. In phenotype assays, BSH and the HypR-controlled disulfide reductase MerA were required for protection against ROS produced under AGXX® stress in *S. aureus*. Altogether, our study revealed a strong thiol-reactive mode of action of AGXX® in *S. aureus* USA300 resulting in an increased BSH redox potential and protein *S*-bacillithiolation.

## Introduction

*Staphylococcus aureus* inhabitants the skin and the nose of one quarter of the human population, but can also lead to serious live-threatening infections when the pathogen enters the bloodstream (Foster, 2004). *S. aureus* causes various diseases ranging from local skin abscesses to systemic and chronic infections, such as septicemia, endocarditis, pneumonia and osteomyelitis. *S. aureus* cells can also form biofilms on medical devices, such as catheters or implants (Archer, 1998; Lowy, 1998; Boucher and Corey, 2008). Multiple antibiotic resistant *S. aureus* isolates are an increasing health-burden that arise in hospitals and in the community, including methicillin-resistant *S. aureus* (MRSA) isolates (Livermore, 2000). *S. aureus* belongs to the most serious pathogens worldwide and is classified together with six Gram-negative bacteria as ESKAPE pathogen by the “European Center of Disease Prevention and Control” (Pendleton et al., 2013). Since treatment option for multiple antibiotic resistant *S. aureus* isolates are limited, new targets for antibiotics need to be identified and alternative antimicrobial approaches developed to combat the increasing problem of antimicrobial resistance. Among these strategies, reactive oxygen species (ROS)-producing antibiotics and inhibitors of antioxidant responses that affect the bacterial redox balance are attractive alternatives as previously shown for other human pathogens (Padiadpu et al., 2016; Tung et al., 2018).

Metals, like silver, copper, and iron are known to cause reactive oxygen species (ROS), such as the highly reactive hydroxyl radical which leads to oxidation of proteins, DNA bases and lipids explaining some antimicrobial effects of metal ions (Grass et al., 2011; Maillard and Hartemann, 2013). Moreover, silver ions can bind to thiol groups of metal-containing proteins, such as copper transporters, metal-sensing regulators or respiratory chain complexes, leading to loss of protein functions. The inhibition of respiratory enzymes in the electron chain might further promote ROS production, such as superoxide anions. The bactericidal effect of metallic copper and silver surfaces was also termed as “contact killing” (Grass et al., 2011; Maillard and Hartemann, 2013; Villapún et al., 2016). These metals are used as surface coating of medical devices, for wound healing and in water pipelines to clean drinking water (Lansdown, 2010). Especially silver has been applied for a long time as medicinal product against wound infections until the evolution of silver resistant bacteria in 1975 (Gupta et al., 1999).

The novel antimicrobial coating AGXX® (Largetec GmbH, Berlin) was recently developed as promising broad-spectrum antimicrobial that can be electroplated on various surfaces, including steel, ceramics, glass and organic polymers (Guridi et al., 2015; Clauss-Lendzian et al., 2018; Vaishampayan et al., 2018). AGXX® strongly inhibited the growth and biofilm formation of many antibiotic resistant nosocomial pathogens, such as *S. aureus, Staphylococcus epidermidis, Enterococcus faecalis*, and *Enterococcus faecium* (Guridi et al., 2015). Furthermore, AGXX® had a strong killing effect on *Legionella erythra* in water pipelines and *Escherichia coli* in urine samples. This antimicrobial effect of AGXX® was much more efficient compared to classical silver (Guridi et al., 2015). AGXX® is composed of two transition metals, silver (Ag) and ruthenium (Ru) that are conditioned with ascorbic acid. The Ag^+^ and Ru^+^ metal ions form a micro-galvanic cell and lead to ROS formation, such as H_2_O_2_ and the strong oxidant hydroxyl radical, which are proposed as main antimicrobial mechanism (Gupta et al., 1999; Guridi et al., 2015; Heiss et al., 2017; Clauss-Lendzian et al., 2018). Using RNA-seq transcriptomics, the antimicrobial mode of action of AGXX® has been recently studied in the MRSA isolate 04-02981 (Vaishampayan et al., 2018) and in a clinical *E. faecalis* isolate (Clauss-Lendzian et al., 2018). Gene expression profiling revealed repression of genes for biofilm formation and virulence factors as well as the *agr* quorum sensing system in *S. aureus*. The induction of the heat-shock regulons HrcA and CtsR was indicative of protein damage in both bacteria (Clauss-Lendzian et al., 2018; Vaishampayan et al., 2018). However, it is not known whether AGXX® stress causes an oxidative stress response and affects the redox state in *S. aureus* due to the production of ROS and if there are potential protection mechanisms against the antimicrobial AGXX®.

*Staphylococcus aureus* is frequently exposed to ROS, such as H_2_O_2_ and the strong oxidant hypochlorous acid (HOCl) during infection by activated macrophages and neutrophils (Winterbourn and Kettle, 2013; Hillion and Antelmann, 2015; Beavers and Skaar, 2016; Winterbourn et al., 2016). Several protection mechanisms are utilized by *S. aureus* to cope with ROS and HOCl, including the low molecular weight (LMW) thiol bacillithiol (BSH) which is important under macrophage infections (Newton et al., 2012; Pöther et al., 2013; Posada et al., 2014; Chandrangsu et al., 2018). BSH is involved in detoxification of various reactive species, ROS, HOCl, electrophiles, toxins and antibiotics and serves as thiol-cofactor for many redox enzymes, such as thiol-*S*-transferases, glyoxalases and antioxidant enzymes (Chandrangsu et al., 2018). Under conditions of HOCl stress, BSH forms mixed disulfides with proteins, termed as protein *S*-bacillithiolation (Chi et al., 2011, 2013; Imber et al., 2018a). Protein *S*-bacillithiolations are widespread redox-modifications, which function in thiol-protection and redox regulation of redox-sensing transcription factors and metabolic enzymes, such as the glycolytic glyceraldehyde-3-phosphate dehydrogenase (GapDH) and the aldehyde dehydrogenase AldA (Chi et al., 2011, 2013; Loi et al., 2015; Imber et al., 2018a,b,c). GapDH was identified as most abundant *S*-bacillithiolated protein in the *S. aureus* redox proteome. GapDH activity was inhibited by *S*-bacillithiolation under H_2_O_2_ and NaOCl stress *in vitro* which was faster compared to its irreversible inactivation by overoxidation (Imber et al., 2018a). Thus, our data confirmed that *S*-bacillithiolation efficiently protects the active sites of redox-sensitive enzymes against overoxidation to Cys sulfonic acids (Loi et al., 2015; Imber et al., 2018c).

Further protection mechanisms against oxidative stress are regulated by the SarA/MarR-family redox-sensing regulators (SarA, MgrA and SarZ), by the HOCl-inducible Rrf2-family regulater HypR and by the peroxide regulon repressor PerR (Horsburgh et al., 2001; Chen et al., 2006, 2009, 2011; Hillion and Antelmann, 2015; Ji et al., 2015; Chandrangsu et al., 2018). The SarZ-controlled OhrA peroxiredoxin is involved in detoxification of organic hydroperoxides that are derived from fatty acid oxidation (Dubbs and Mongkolsuk, 2007). The OhrR-homologs SarZ and MgrA can be regulated by *S*-thiolation with a synthetic LMW thiol, resulting in derepression of transcription of their target genes under oxidative stress (Abomoelak et al., 2009; Poor et al., 2009; Chen et al., 2011; Sun et al., 2012; Hillion and Antelmann, 2015). Recent OxICAT analyses revealed increased oxidation of the redox-sensing Cys residues of MgrA and SarZ under HOCl stress (Imber et al., 2018a). This indicates that both regulators could be regulated by *S*-bacillithiolation in *S. aureus* under HOCl stress.

The novel redox-sensing regulator HypR was shown to control the pyridine nucleotide disulfide reductase MerA which provides specific protection under HOCl stress and macrophage infections (Loi et al., 2018). HypR belong to the Rrf2-family that senses and responds to HOCl stress by a thiol-redox switch involving Cys33-Cys99′ intersubunit disulfide formation between adjacent subunits of the HypR dimer (Loi et al., 2018). In addition, the H_2_O_2_ response is controlled by the peroxide repressor PerR in *S. aureus*. PerR uses metal-catalyzed histidine oxidation to sense peroxide stress (Horsburgh et al., 2001; Lee and Helmann, 2006; Ji et al., 2015; Pinochet-Barros and Helmann, 2018). PerR regulates genes encoding catalases (KatA), peroxiredoxins (AhpCF, Bcp, Tpx), the miniferritin (Dps) and the iron-sulfur cluster SUF machinery under H_2_O_2_ stress in *S. aureus* (Horsburgh et al., 2001; Ji et al., 2015; Pinochet-Barros and Helmann, 2018).

In this study, we applied RNA-seq transcriptomics to study the mode of action of the AGXX® antimicrobial coating in *S. aureus* USA300. Our results revealed that AGXX® causes a thiol-specific oxidative stress response, as shown by the strong up-regulation of the PerR, HypR, QsrR, MhqR, and CstR regulons. The up-regulation of the CtsR and HrcA heat shock regulons is caused by oxidative protein damage and aggregation. The induction of the GraRS and SigmaB regulons further indicates cell wall and general stress responses under AGXX® exposure. Finally, AGXX® stress interferes with metal homeostasis as revealed by the increased transcription of the CsoR, Fur, and Zur regulons. AGXX® further leads to oxidative shift in the BSH redox potential, enhanced protein *S*-bacillithiolation and protein aggregation, supporting its thiol-reactive mode of action. Phenotype assays revealed that the LMW thiol BSH and the HypR-controlled disulfide reductase MerA provide protection under AGXX® stress in *S. aureus*.

## Materials and Methods

### Preparation of AGXX® Microparticles

AGXX® microparticles of charge 373 were kindly provided by Largentec GmbH, Berlin, Germany. The AGXX® microparticles consist of a powder of silver with particle size of 1.5–2.5 μm (MaTecK, Germany), which was chemically coated with ruthenium (Guridi et al., 2015; Heiss et al., 2017). The coating was performed in alkaline medium where the Ru(III) ions were first oxidized by NaOCl to RuO_4_. Reduction of RuO_4_ to Ru or RuO_X_ was performed by addition of NaNO_2_ as described previously (Chen et al., 2010). The black AGXX® powder was further conditioned with 50 mM ascorbic acid for 2 h, followed by filtration, washing with deionized water and drying.

### Bacterial Strains, Growth and Cultivation

The *S. aureus* strains used for the experiments with AGXX® include the highly virulent community-acquired (CA) MRSA strain *S. aureus* USA300 and the hospital-acquired (HA) MRSA strain *S. aureus* COL. For phenotype analysis we further compared the *S. aureus* USA300 Δ*bshA* mutant (Posada et al., 2014), the *S. aureus* COL Δ*merA* deletion mutant and its complemented strain as described previously (Loi et al., 2018). *S. aureus* strains were cultivated in RPMI medium as described (Imber et al., 2018a). For survival phenotype assays under AGXX® stress, *S. aureus* USA300 was grown in RPMI medium to an OD_500_ of 0.5 and exposed to different amounts of 3–10 μg/ml AGXX® microparticles. The culture was diluted and 10 μl of serial dilutions were spotted onto LB agar plates. Colony forming units (CFU) were observed after incubation of the LB plates for 24 h.

### Live/Dead Viability Assays

The viability assay of *S. aureus* USA300 was performed after treatment with sub-lethal and lethal amounts (4 and 10 μg/ml) of AGXX® at an OD_500_ of 0.5 using the LIVE/DEAD™ BacLight™ Bacterial Viability Kit (Thermo Fisher) according to the instructions of the manufacturers. *S. aureus* strains were stained with SYTO9 and propidium iodide for each 15 min. Stained cells were washed in phosphate buffer and live or dead cells were visualized using a fluorescence microscope (Nikon, Eclipse, T*i*2) (SYTO9 Ex: 488 nm, propidium iodide Ex: 555 nm). Fluorescence intensity was measured after excitation at 488 and 555 nm and false-colored in green or red for live or dead cells, respectively.

### RNA Isolation, Library Preparation, and Next Generation cDNA Sequencing

*Staphylococcus aureus* USA300 was cultivated in RPMI medium in 3 biological replicates and treated with 4.5 μg/ml AGXX® stress as described previously (Loi et al., 2017). *S. aureus* cells were harvested before (as untreated control) and 30 min after exposure to 4.5 μg/ml AGXX® and disrupted in 3 mM EDTA/ 200 mM NaCl lysis buffer with a Precellys24 ribolyzer. RNA isolation was performed using the acid phenol extraction protocol as described (Chi et al., 2014). The RNA quality was checked by Trinean Xpose (Gentbrugge, Belgium) and the Agilent RNA Nano 6,000 kit using an Agilent 2,100 Bioanalyzer (Agilent Technologies, Böblingen, Germany). Ribo-Zero rRNA Removal Kit (Bacteria) from Illumina (San Diego, CA, USA) was used to remove the rRNA. TruSeq Stranded mRNA Library Prep Kit from Illumina (San Diego, CA, United States) was applied to prepare the cDNA libraries. The cDNAs were sequenced paired end on an Illumina HiSeq 1,500 (San Diego, CA, United States) using 70 and 75 bp read length and a minimum sequencing depth of 10 million reads per library. The transcriptome sequencing raw data files are available in the ArrayExpress database (www.ebi.ac.uk/arrayexpress) under accession number E-MTAB-7074.

### Bioinformatics Data Analysis, Read Mapping, Data Visualization, and Analysis of Differential Gene Expression

The paired end cDNA reads were mapped to the *S. aureus* USA300_TCH1516 genome sequence (accession number CP000730) (Highlander et al., 2007) using bowtie2 v2.2.7 (Langmead and Salzberg, 2012) with default settings for paired-end read mapping. All mapped sequence data were converted from SAM to BAM format with SAMtools v1.3 (Li et al., 2009) and imported to the software ReadXplorer v.2.2 (Hilker et al., 2016).

Differential gene expression analysis of triplicates including normalization was performed using Bioconductor package DESeq2 (Love et al., 2014) included in the ReadXplorer v2.2 software (Hilker et al., 2016). The signal intensity value (A-value) was calculated by log2 mean of normalized read counts and the signal intensity ratio (M-value) by log2 fold-change. The evaluation of the differential RNA-seq data was performed using an adjusted *p*-value cut-off of *P* ≤ 0.05 and a signal intensity ratio (*M*-value) cut-off of ≥ 0.6 or ≤ −0.6 (fold-change of ± 1.5). The cut-off was determined as ± 0.6 since the majority of differentially transcribed regulons fall in this range that are related to the redox-active mode of action of AGXX®.

### Construction of the Voronoi Transcriptome Treemap

The Paver software (DECODON GmbH, Greifswald, Germany) was used to generate the AGXX® transcriptome treemap as previously described (Mehlan et al., 2013), showing log2-fold changes (M-values) of selected genes, operons and regulons that are up-regulated under AGXX® stress. The treemap is structured into regulons (white labels), genes and operons (smaller labels). The cell sizes are defined as absolute log2-fold changes of expression levels after AGXX® stress divided by the untreated control. The log2-fold ratios of AGXX® vs. the untreated controls are displayed by a blue-red color gradient where blue indicates repression and red induction under AGXX ® stress.

### Northern Blot Experiments

Northern blot analysis was performed as described previously (Wetzstein et al., 1992) using RNA isolated from *S. aureus* USA300 that was exposed to 4.5 μg/ml AGXX® for 30 min as indicated. Hybridizations were performed using the digoxigenin-labeled *merA*-specific antisense RNA synthesized *in vitro* using T7 RNA polymerase as described (Tam Le et al., 2006; Loi et al., 2018).

### Western Blot Analysis

*Staphylococcus aureus* USA300 cells were grown in LB until an OD_540_ of 2 and harvested by centrifugation. Cells were transferred to Belitsky minimal medium (BMM) for 1 h and treated with increasing amounts of 3–10 μg/ml AGXX® microparticles for 30 min. Cells were harvested and washed in TE-buffer (pH 8.0) with 50 mM NEM, disrupted using the ribolyzer and the protein extract was cleared from cell debris by centrifugation. Protein amounts of 25 μg were separated using 12% SDS-PAGE and subjected to BSH-specific Western blot analysis using the polyclonal rabbit anti-BSH antiserum as described previously (Chi et al., 2013).

### Measurement of the BSH Redox Potential in *S. aureus* COL Under AGXX® Stress Using the Brx-roGFP2 Biosensor

For BSH redox potential measurements under AGXX® stress using the Brx-roGFP2 biosensor, we applied *S. aureus* COL since biosensor expression and fluorescence intensity was higher in *S. aureus* COL compared to *S. aureus* USA300 as revealed previously (Loi et al., 2017). *S. aureus* COL expressing the Brx-roGFP2 plasmid (Loi et al., 2017) was grown in RPMI medium to OD_500_ of 0.5 and exposed to 4 μg/ml of AGXX® for different times. The Brx-roGFP2 redox state was blocked with 10 mM N-ethylmaleimide (NEM) and the *S. aureus* cells transferred to the microplate wells for fluorescence measurements as described (Loi et al., 2017). Samples for fully reduced and oxidized controls were treated for 20 min with 10 mM DTT and 5 mM diamide, respectively. The Brx-roGFP2 biosensor fluorescence emission was measured at 510 nm after excitation at 405 and 488 nm using the CLARIOstar microplate reader (BMG Labtech). The OxD of the Brx-roGFP2 biosensor was determined for each sample and normalized to fully reduced (DTT-treated) and oxidized (diamide-treated) controls as described (Loi et al., 2017) based to the following equation (1):

(1) $$\begin{array}{l} \text{O}\times \text{D}=\frac{{\text{I}405}_{\text{sample }}\times \;{\text{I}488}_{\text{red }}-\;{\text{I}405}_{\text{red }}\times \;{\text{I}488}_{\text{sample}}}{{\text{I}405}_{\text{sample }}\times \;{\text{I}488}_{\text{red }}-\;{\text{I}405}_{\text{sample }}\times \;{\text{I}488}_{\text{ox}}\;+\;{\text{I}405}_{\text{ox }}\times {\text{ I}488}_{\text{sample}}-\;{\text{I}405}_{\text{red }}\times \;{\text{I}488}_{\text{sample}}} \end{array}$$

The values of *I*405_sample_ and *I*488_sample_ are the observed fluorescence excitation intensities at 405 and 488 nm, respectively. The values of *I*405_red_, *I*488_red_, *I*405_ox_, and *I*488_ox_ represent the fluorescence intensities of fully reduced and oxidized controls, respectively.

Based on the OxD and $E_{\text{roGFP2}}^{o^{\prime }}$ = – 280 mV (Dooley et al., 2004), the BSH redox potential can be calculated according to the Nernst equation (2):

(2) $$\begin{array}{l} E_{\text{BSH}}=E_{\text{roGFP2}}=E_{\text{roGFP2}}^{o^{\prime }}-\;\left(\frac{\text{RT}}{\text{2F}}\;\right)*\text{In }\left(\frac{1-\text{O}\times \text{D}}{\text{O}\times \text{D}}\;\right) \end{array}$$

### Analysis of Protein Aggregates After AGXX® Stress

The fractions with intracellular protein aggregates were isolated as described previously with some modifications (Tomoyasu et al., 2001; Groitl et al., 2017). *S. aureus* USA300 was grown in RPMI medium until an OD_500_ of 0.5 and exposed to 4, 8, or 10 μg/ml AGXX® for 30 min. The *S. aureus* samples were adjusted to 8 ml of an OD_500_ of 1 and harvested by centrifugation. The cell pellets were resuspended in 40 μl buffer A (10 mM potassium phosphate pH 6.5, 1 mM EDTA, 20% sucrose, 5 μg/ml lysostaphin) and incubated on ice for 30 min. The cells were further diluted in 360 μl buffer B (10 mM potassium phosphate pH 6.5, 1 mM EDTA) and disrupted with glass beads using the PrecellysEvolution ribolyzer (Bertin technologies). Intact cells were removed by centrifugation at 2,000 rpm and the insoluble protein fraction was collected by a second centrifugation step (14,000 rpm, 45 min). Pellets were frozen at−20°C for 30 min and resuspended in 400 μl of buffer B followed by brief sonication. Membrane proteins were dissolved with 160 μl buffer C (buffer B plus 2% Nonidet P-40) and the protein aggregates collected by centrifugation. The aggregated proteins were washed with 400 μl buffer B and separated using SDS-PAGE analysis.

## Results

### The Novel Antimicrobial Coating AGXX® Efficiently Inhibits the Growth and Viability of *S. aureus* USA300

**Figure 1:**
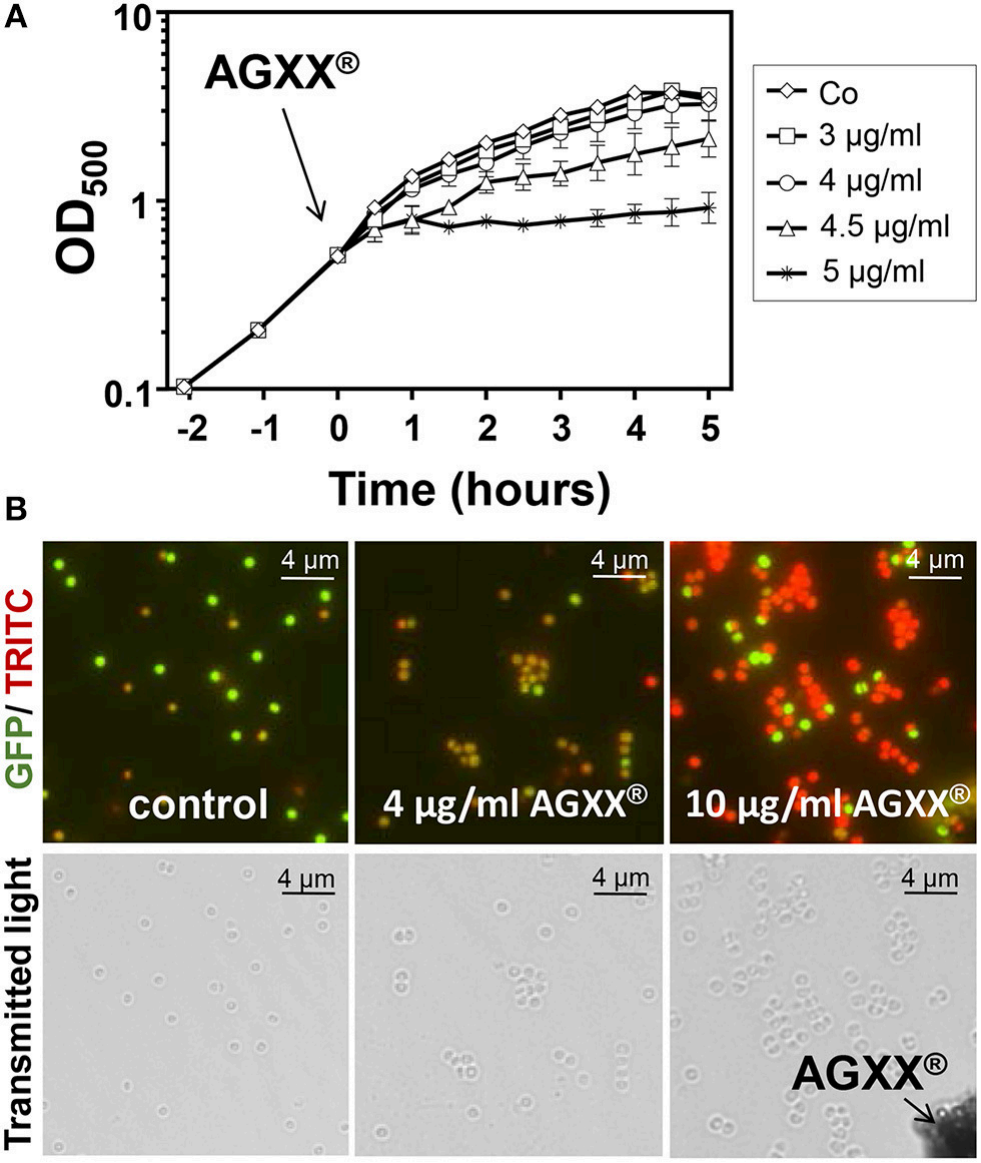
AGXX® has a strong antimicrobial effect on *S. aureus* USA300. **(A)** Growth curves of *S. aureus* USA300 were performed in RPMI medium which was exposed to 3–5 μg/ml AGXX® stress at an OD_500_ of 0.5. The results are from 3 biological replicate experiments. Error bars represent the standard deviation (SD). **(B)** Viability of *S. aureus* USA300 wild type was analyzed before and 30 min after exposure to sub-lethal (4 μg/ml) or lethal amounts (10 μg/ml) of AGXX® at OD_500_ of 0.5 using the LIVE/DEAD™ BacLight™ Bacterial Viability Kit. Live and dead *S. aureus* USA30 cells were visualized with a fluorescence microscope (Nikon, Eclipse, T*i*2). *S. aureus* with an intact cytoplasmic membrane shows green fluorescence and dead cells with damaged membranes exhibit red fluorescence. The black arrow denotes AGXX® microparticles.

To investigate the effective antimicrobial amount of AGXX® microparticles that inhibit the growth of *S. aureus* USA300, we performed growth curves and live/dead assays. *S. aureus* USA300 was grown in RPMI medium and exposed to different amounts of AGXX® microparticles (3–5 μg/ml) during the exponential growth phase. The growth of *S. aureus* USA300 was not affected by 3–4 μg/ml AGXX®, but 4.5–5 μg/ml AGXX® significantly reduced the growth rate (Figure 1A). Live/dead staining with the LIVE/DEAD™ Bacterial Viability Kit was used to analyze bacterial survival after exposure of *S. aureus* to sub-lethal (4 μg/ml) and lethal amounts (10 μg/ml) of AGXX®. The results revealed that 4 μg/ml is sub-lethal for *S. aureus* while 10 μg/ml has a strong killing effect on the majority of *S. aureus* cells in RPMI medium (Figure 1B). These results of live/dead assays were confirmed by analysis of colony forming units (CFUs) as shown in the next sections for phenotype analyses of different mutants. To analyze the physiological effect and stress response caused by AGXX® in the transcriptome of *S. aureus* USA300, we have chosen sub-lethal amounts of 4.5 μg/ml AGXX® which slightly affected the growth rate (Figure 1A).

### AGXX® Stress Causes a Thiol-Specific Oxidative and Metal Stress Response in the *S. aureus* Transcriptome

**Figure 2:**
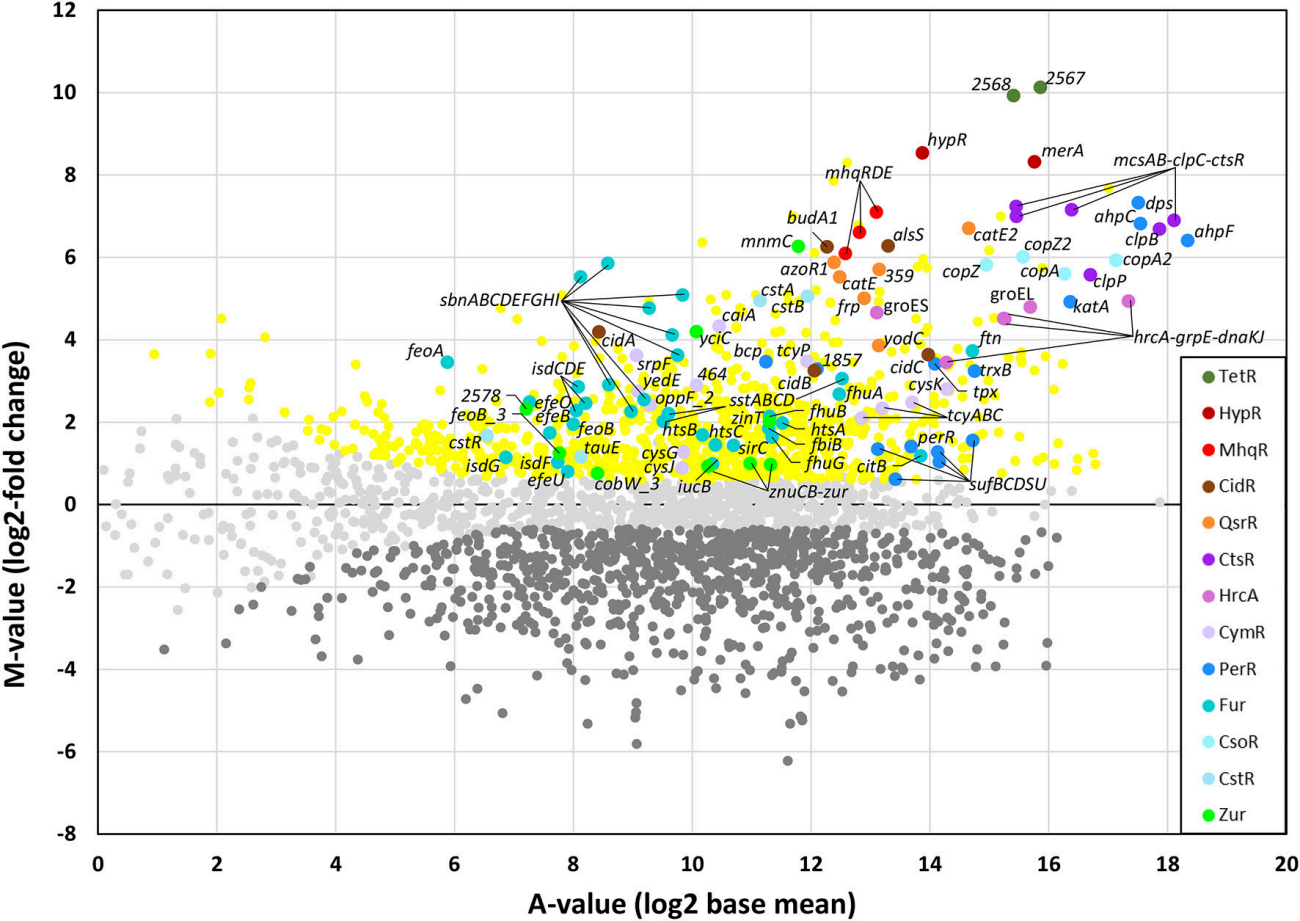
RNA-seq transcriptomics of *S. aureus* USA300 after 30 min of AGXX® stress. For RNA-seq transcriptomics, *S. aureus* USA300 was grown in RPMI medium and treated with 4.5 μg/ml AGXX® for 30 min. The gene expression profile under AGXX® stress is shown as ratio/intensity scatter plot (M/A-plot) which is based on differential gene expression analysis using DeSeq2. Colored symbols indicate significantly induced (red, orange, brown, blue, cyan, violet, green, yellow) or repressed (dark gray) transcripts (*M* ≥0.6 or ≤ -0.6; *P* ≤ 0.05). Light gray symbols denote transcripts with no fold-changes after AGXX® stress (*P* > 0.05). The PerR, TetR, HypR, QsrR, MhqR, CtsR, HrcA, CidR, CymR, Fur, CstR, CsoR, and Zur regulons are most strongly up-regulated under AGXX® stress. The transcriptome analysis was performed from three biological replicates. The RNA-seq expression data of all genes after AGXX® stress and their regulon classifications are listed in Tables S1, S2.

**Figure 3:**
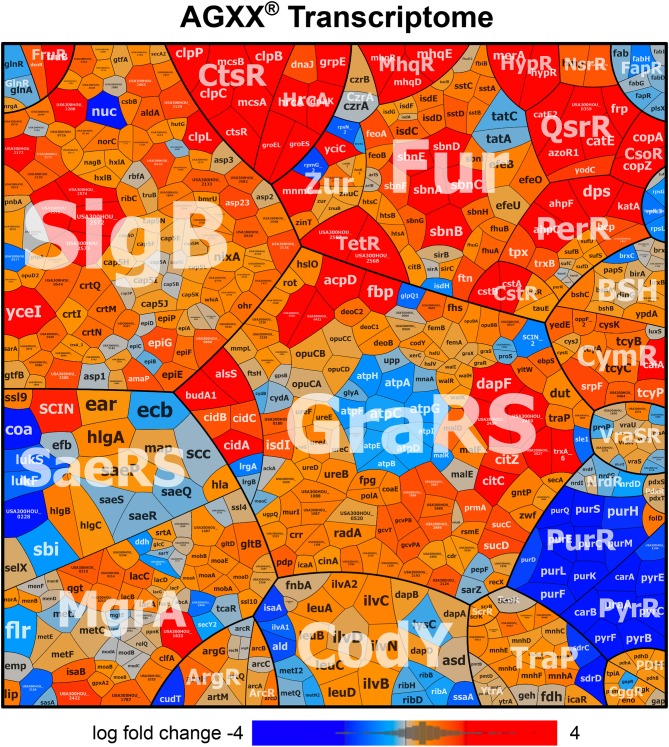
AGXX® induces a strong thiol-specific oxidative and metal stress response in the RNA-seq transcriptome of *S. aureus* USA300. The treemap shows the log2-fold changes (M-values) for the induction and repression of selected regulons after exposure to 4.5 μg/ml AGXX® stress in the RNA-seq transcriptome of *S. aureus* USA300. The genes are classified into operons and regulons based on the RegPrecise database (http://regprecise.lbl.gov/RegPrecise/index.jsp) and previous studies (Mäder et al., 2016). Log2-fold changes in gene expression are visualized using a red-blue color code where red indicates log2-fold induction and blue repression of transcription under AGXX® exposure. AGXX® caused a strong thiol-specific oxidative and metal stress response and protein damage as shown by the strong induction of the PerR, HypR, QsrR, MhqR, CtsR, HrcA, and CstR regulons in *S. aureus* USA300. The induction of the CsoR, Fur, Zur, SigmaB, and GraRS regulons further indicates metal stress, cell wall and general stress responses in *S. aureus*. The detailed transcriptome data of all differentially expressed genes in response to AGXX® stress are presented in Tables S1, S2.

To investigate the stress response caused by AGXX® in *S. aureus* USA300, we studied the changes in the RNA-seq transcriptome after exposure to sub-lethal amounts of 4.5 μg/ml AGXX® stress. The RNA samples were isolated from 3 biological replicates before (control) and 30 min after AGXX® stress and analyzed by RNA-seq transcriptomics as in previous studies (Hillion et al., 2017; Loi et al., 2018). For significant expression changes, the M-value cut-off (log2-fold change AGXX®/control) of ≥0.6 and ≤ −0.6 (fold-change of ± 1.5, *P* ≤ 0.05) was chosen since the majority of most strongly induced regulons fall in this range. In total, 925 transcripts were significantly >1.5-fold up-regulated and 896 were <-1.5-fold down-regulated in the transcriptome of *S. aureus* under AGXX® stress (Figure 2 and Tables S1, S2). Among the up-regulated genes are 149 with fold-changes of >10-fold that could be classified into the PerR, HypR, QsrR, MhqR, CtsR, HrcA, CidR, CymR, CstR, CsoR, Fur, Zur, SigB, and GraRS regulons. The most strongly induced regulons in the AGXX® transcriptome are shown in the ratio/intensity scatter plot (M/A-plot) and visualized using the Voronoi transcriptomics treemap by red-blue color codes (Figures 2, 3 and Table S2).

We have previously used RNA-seq transcriptomics to analyze the response of *S. aureus* USA300 to the strong oxidant NaOCl (Loi et al., 2018). NaOCl stress caused a thiol-specific oxidative stress signature and protein damage in *S. aureus* USA300 as revealed by the induction of the HypR, PerR, QsrR, HrcA, and CtsR regulons (Loi et al., 2018). NaOCl stress further provoked the weak induction of the Fur and Zur regulons in *S. aureus* indicative of metal starvation. Treatment of *S. aureus* USA300 with 4.5 μg/ml AGXX® stress resulted in similar changes in gene expression, with increased expression of the PerR, HypR and QsrR regulons that are related to the thiol-specific oxidative and electrophile stress response (Figures 2, 3). The HypR-controlled *hypR-merA* operon was among the most strongly up-regulated hits (fold-changes of 320-372). The *USA300HOU_2567-68* operon of unknown function was even 977-1117-fold induced under AGXX® stress. The *USA300HOU_2567-68* operon is regulated by the upstream located *USA300HOU_2566* gene that encodes for a TetR-family regulator (our unpublished data). Thus, the *USA300HOU_2567-68* operon was renamed as TetR regulon.

In addition, the CtsR and HrcA regulons were highly up-regulated under AGXX® with fold-changes of >119 for the *ctsR-mcsA-mcsB-clpC* operon and of >30 for the *hrcA-grpE-dnaKJ* operon. The HrcA and CtsR repressors control the transcription of proteases and chaperones of the quality control machinery (Frees et al., 2007, 2014), indicating that AGXX® stress might cause strong protein damage and aggregation. The increased transcription of the Fur and Zur regulons under AGXX® stress further indicates iron and zinc depletion under AGXX® stress, confirming its impact on metal homeostasis. Moreover, in agreement with previous studies, the Cu^+^ sensing CsoR-regulated *copZA* operon was highly upregulated (48–56-fold) under AGXX® stress in *S. aureus* (Grossoehme et al., 2011). In addition, the CsoR-like persulfide-specific CstR regulon which includes the *cstAB-sqr* and *cstR-tauE* operons was strongly induced under AGXX® stress in *S. aureus* (fold-changes of >30 and 2–3, respectively). The CstR regulon responds to hydrogen sulfide (H_2_S) and nitroxyl (HNO) (Luebke et al., 2013, 2014; Peng et al., 2017). AGXX® might cause thiol-oxidation of the metal binding Cys ligands of CsoR, CstR, Fur and Zur that could trigger the inactivation of the repressors and derepression of the corresponding regulons. We further noted the up-regulation of the CymR regulon and genes related to bacillithiol (BSH) biosynthesis and recycling (e.g., *bshA* operon, *bshB, bshC, brxB*, and *ypdA*) under AGXX® stress in *S. aureus*. This supports the notion that AGXX® leads to increased thiol-oxidation in proteins and depletion of the LMW thiol BSH.

The SigmaB regulon was also induced under AGXX® stress in *S. aureus*, which was required for intracellular growth during cell culture infection assays (Pförtner et al., 2014). The cell wall stress responsive GraRS is further induced under AGXX® stress, which responds to cell-wall active antibiotics and antimicrobial peptides and confers resistance to oxidative stress in *S. aureus* (Falord et al., 2011). However, in contrast to previous studies with AGXX® surface coating, we did not find significant changes in expression of the AgrAC two-component quorum-sensing system controlling virulence factors, such as hemolysins and surface factors (Novick, 2003; Queck et al., 2008). In our RNA-seq analysis, sub-lethal amounts of AGXX® microparticles caused a strong thiol-specific oxidative and metal stress response as well as protein damage as major transcriptome signature.

### AGXX® Stress Causes Increased Protein *S*-bacillithiolation, Protein Aggregation and an Oxidative Shift in the BSH Redox Potential in *S. aureus*

**Figure 4:**
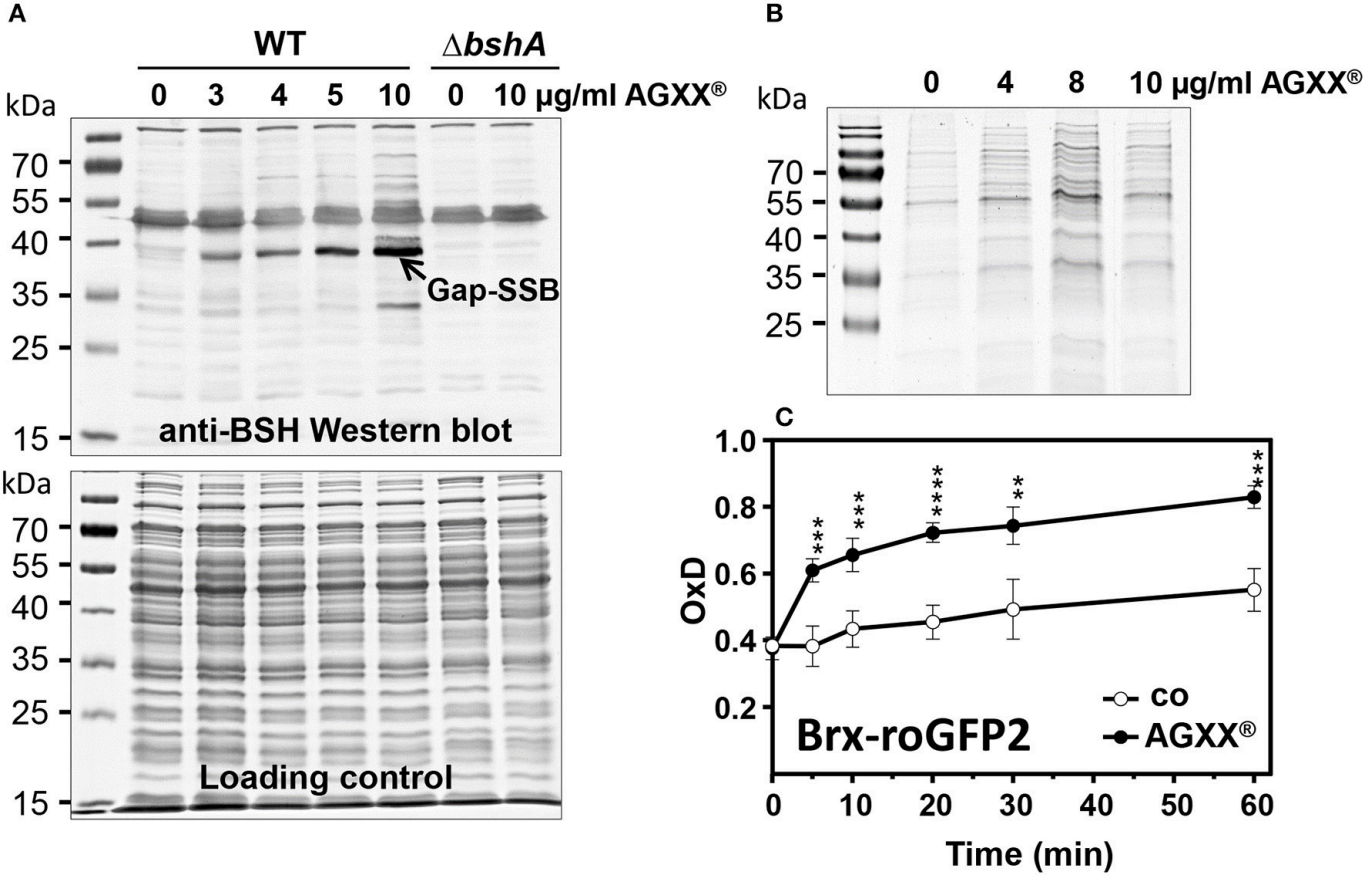
AGXX® causes protein *S*-bacillithiolation, protein aggregation and an oxidative shift in the BSH redox potential in *S. aureus*. **(A)** Non-reducing BSH-specific Western blot analysis showed increasing of *S*-bacillithiolated GapDH protein in *S. aureus* USA300 strain under AGXX® stress. The Coomassie-stained SDS-PAGE is shown as loading control below the BSH Western blot. **(B)** To analyze protein aggregates, *S. aureus* cells were grown in RPMI medium to OD_500_ of 0.5 and treated with 4–10 μg/ml AGXX® for 30 min. The insoluble protein fractions of protein aggregates were obtained by centrifugation of the cell lysates and repeated centrifugation of the NP-40-dissolved pellet as described in the Methods section. The insoluble protein fractions were analyzed by SDS-PAGE and visualized with Coomassie staining **(C)** *S. aureus* COL expressing Brx-roGFP2 was treated with the sub-lethal amount of 4 μg/ml AGXX® at OD_500_ of 0.5 in RPMI medium and the biosensor oxidation degree (OxD) was measured at different times after stress. The Brx-roGFP2 biosensor is rapidly oxidized by AGXX®. The OxD values of the Brx-roGFP2 biosensor measurements with and without AGXX® were calibrated to fully reduced (DTT-treated) and oxidized (diamide-treated) control samples. The OxD values of fully reduced and oxidized controls were defined as “0” and “1,” respectively. The data were calculated as mean values from 3 biological replicate experiments. Error bars represent the SD and *p*-values are calculated using a Student's unpaired two-tailed *t*-test by the graph prism software. Symbols are defined as follows: **p ≤ 0.01; ***p ≤ 0.001 and ****p ≤ 0.0001 (*p* < 0.0003 for control/AGXX stress at 5 min; *p* = 0.0002 at 10 min; *p* < 0.0001 at 20 min; *p* = 0.0013 at 30 min; *p* = 0.0001 at 60 min).

NaOCl stress has been previously shown to cause increased thiol-oxidation of 58 Cys residues in the redox proteome of *S. aureus* USA300 (Imber et al., 2018a). Among those NaOCl-sensitive proteins are five *S*-bacillithiolated proteins including the glycolytic GapDH as major target for *S*-bacillithiolation (Imber et al., 2018a). Since the transcriptome signature overlaps between NaOCl and AGXX® stress, we hypothesized that AGXX® could also lead to increased *S*-bacillithiolation in *S. aureus* USA300. Using BSH-specific non-reducing Western blots, we monitored the level of *S*-bacillithiolated GapDH after exposure of *S. aureus* USA300 to different amounts of 3–10 μg/ml AGXX® (Figure 4A). As control, the *bshA* mutant was exposed to 10 μg/ml AGXX® which should not show any BSH-mixed disulfides in the BSH-Western blot. The treatment of *S. aureus* USA300 with sub-lethal 3–4 μg/ml AGXX® resulted in increased levels of the *S*-bacillithiolated GapDH (GapDH-SSB). The level of GapDH-SSB gradually increased with higher AGXX® amounts and bands of other *S*-bacillithiolated proteins appeared with 10 μg/ml AGXX® (Figure 4A). This confirms a similar thiol-reactive nature of AGXX® and NaOCl, which both induce protein *S*-bacillithiolation in *S. aureus* USA300.

The strong induction of the HrcA and CtsR regulons further indicates oxidative protein unfolding and aggregation which has been shown under HOCl stress previously (Groitl et al., 2017). Thus, we isolated the insoluble protein fraction that includes protein aggregates after exposure of *S. aureus* USA300 to AGXX® stress. Increasing levels of 4–10 μg/ml AGXX® microparticles resulted in elevated amounts of aggregated proteins as shown in the SDS-PAGE of the insoluble protein fractions (Figure 4B). Thus, AGXX® clearly caused oxidative protein unfolding and aggregation in *S. aureus* USA300.

The increased level of protein *S*-bacillithiolation of GapDH under AGXX® stress points to an oxidative shift in the BSH redox potential (*E*_BSH_) in *S. aureus* USA300. We have recently constructed a genetically encoded Brx-roGFP2 biosensor to measure dynamic *E*_BSH_ changes in *S. aureus* (Loi et al., 2017). Using the Brx-roGFP2 biosensor, we analyzed the changes in *E*_BSH_ in *S. aureus* COL after different times of treatment with sub-lethal 4 μg/ml AGXX® (Figure 4C). The oxidation degree (OxD) of the biosensor was around 0.4 in *S. aureus* control cells at an OD_500_ of 0.5 and increased to an OxD of ~0.5 after 60 min. In AGXX®-treated cells, the OxD of the Brx-roGFP2 biosensor increased to 0.6–0.7 after 5–20 min and reached nearly the fully oxidized state of 0.8 after 60 min of stress (Figure 4C). This confirms the thiol-reactive mode of action of AGXX® which leads to an oxidative shift of *E*_BSH_ due to increased ROS formation as revealed previously (Clauss-Lendzian et al., 2018).

### Bacillithiol and the HypR-controlled Disulfide Reductase MerA Provide Protection Under Oxidative Stress Generated During AGXX® Stress in *S. aureus* USA300

**Figure 5:**
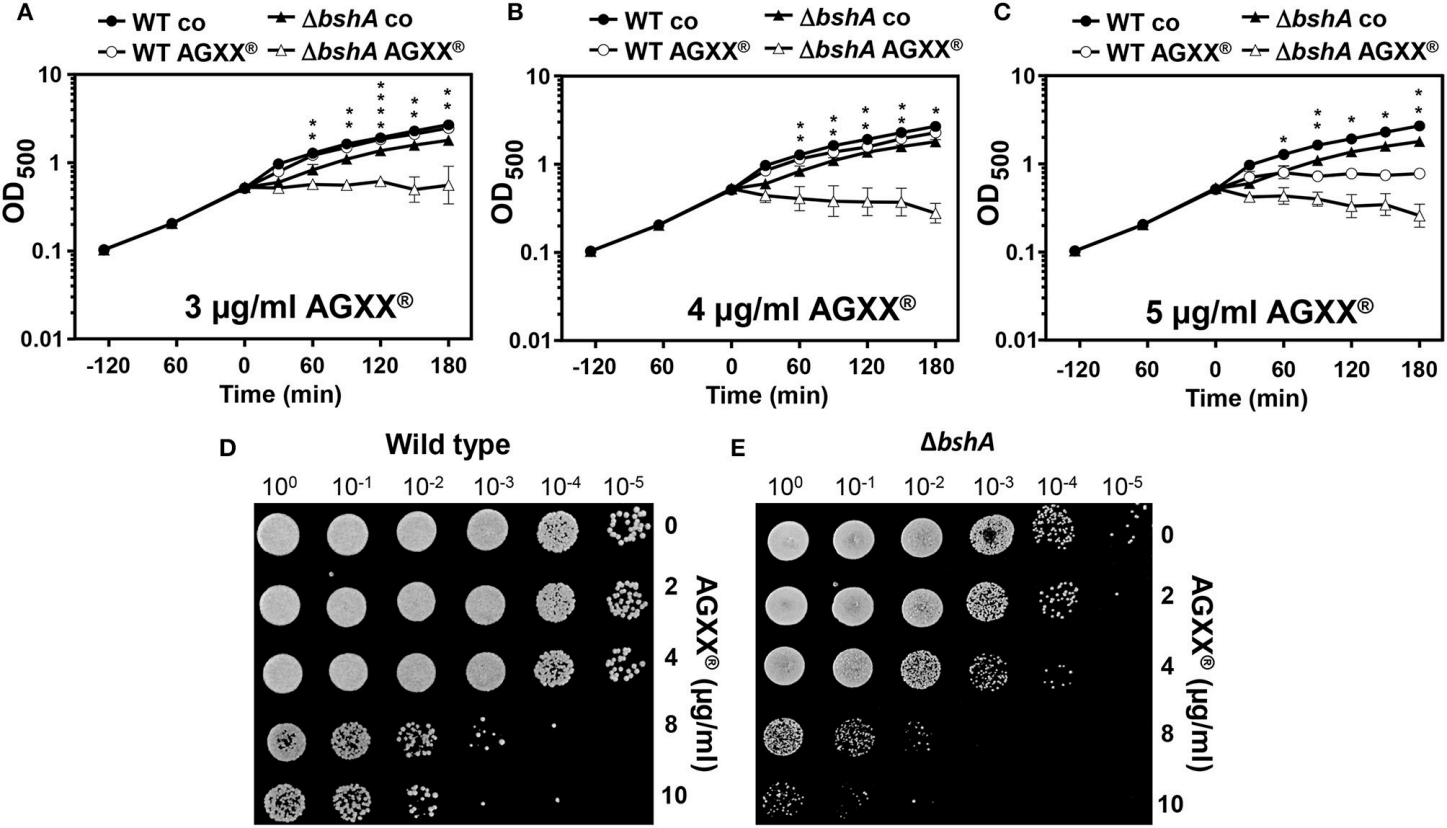
BSH protects *S. aureus* against AGXX® stress. Growth phenotype analyses **(A–C)** and survival assays **(D–E)** were performed for the *S. aureus* USA300 wild type (WT) and the *bshA* mutant before and after exposure to AGXX® stress. **(A–C)** For growth comparisons, strains were exposed to 3, 4, and 5 μg/ml AGXX® stress at an OD_500_ of 0.5. The results are from 3 biological replicate experiments. Error bars represent the SD and the statistics was calculated using a Student's unpaired two-tailed *t*-test by the graph prism software. Symbols are defined as follows:*p ≤ 0.05; **p ≤ 0.01 and ****p ≤ 0.0001. **(D–E)** For survival assays, *S. aureus* USA300 wild-type **(D)** and the *bshA* mutant **(E)** were grown in RPMI until an OD_500_ of 0.5 and treated with 2–10 μg/ml AGXX®. Survival assays were performed by spotting 10 μl of serial dilutions after 2 and 3 h of AGXX® exposure onto LB agar plates to observe CFUs after 24 h incubation.

The RNA-seq transcriptome results and redox biosensor measurements revealed the thiol-reactive mode of action of AGXX® in *S. aureus*. Next, we studied the impact of the LMW thiol BSH in protection against ROS produced under AGXX® stress in *S. aureus*. We compared the growth and survival of the *S. aureus* USA300 wild type and the *bshA* mutant after treatment with increasing doses of AGXX®. While the growth of the wild type was not affected with 3–4 μg/ml AGXX®, the *bshA* mutant was very sensitive and showed a growth defect with 3 μg/ml AGXX® (Figures 5A–C). The survival of the *bshA* mutant was also strongly decreased after exposure to 4 μg/ml which is sub-lethal for the wild type. In addition, lethal doses of 8–10 μg/ml AGXX® have a stronger killing effect for the *bshA* mutant compared to the wild type (Figures 5D,E). These phenotype results indicate that BSH provides protection under AGXX® stress and is most likely involved in detoxification of ROS, such as H_2_O_2_ and hydroxyl radical generated during AGXX® exposure in *S. aureus*.

**Figure 6:**
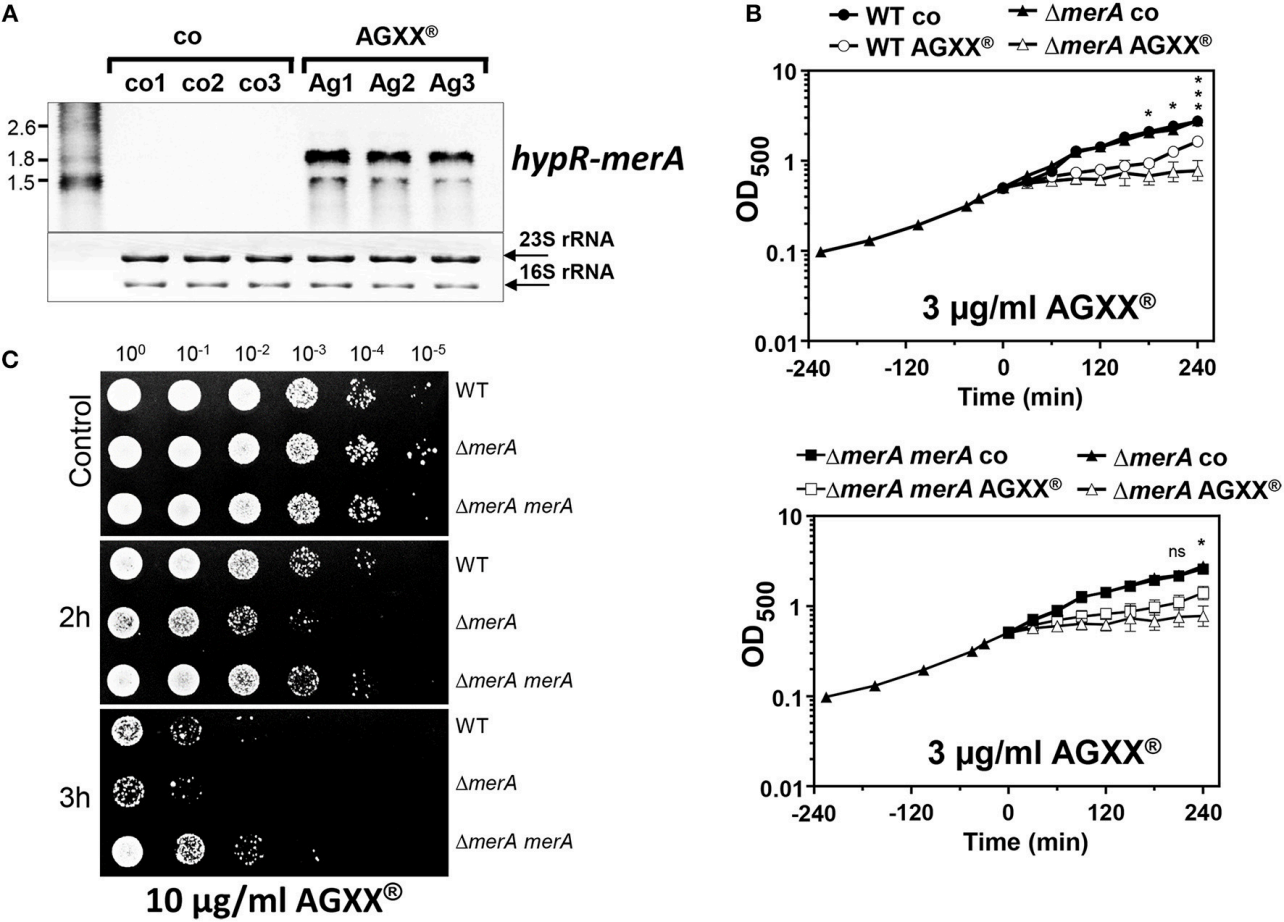
The NADPH-dependent disulfide reductase MerA is strongly induced and required for growth and survival under AGXX® stress in *S. aureus*. **(A)** Northern blot analysis was performed for *hypR-merA* operon transcription in *S. aureus* USA300 wild type before (Co1-3) and 30 min after exposure to 4.5 μg/ml AGXX® (Ag1-3). The RNA samples 1–3 correspond to 3 biological replicates. The methylene blue bands denote the 16S and 23S rRNAs as RNA loading controls below the Northern blots. **(B)** The growth curves were compared for the *S. aureus* COL wild type (WT), the *merA* deletion mutant and the *merA* complemented strain in RPMI medium before (control) and after treatment with 3 μg/ml AGXX®. The time point before AGXX® exposure was set to “0.” Results of three biological replicates are presented as mean values. Error bars represent the SD and the statistics was calculated using a Student's unpaired two-tailed *t*-test by the graph prism software. Symbols are defined as follows: ^ns^*p* > 0.05; *p ≤ 0.05 and ***p ≤ 0.001. **(C)** Survival was analyzed for *S. aureus* COL wild-type (WT), the *merA* mutant and the complemented strain after exposure to 10 μg/ml AGXX® in RPMI medium. Survival assays were performed by spotting 10 μl of serial dilutions after 2 and 3 h of AGXX® exposure onto LB agar plates. Three biological replicate experiments were performed and one representative survival experiment is shown.

In addition, the *hypR-merA* operon was most strongly induced under AGXX® stress (fold-changes of 320–372) in the RNA-seq transcriptome, which was previously characterized as defense mechanism under HOCl stress in *S. aureus* (Loi et al., 2018). The Rrf2 family redox regulator HypR was shown to sense HOCl stress by a thiol-disulfide switch leading to repressor inactivation and induction of the NADPH-dependent flavin disulfide reductase MerA. MerA provides protection under HOCl stress and infection conditions in *S. aureus* COL (Loi et al., 2018). The strong induction of *hypR-merA* operon in *S. aureus* by sub-lethal 4.5 μg/ml AGXX® was confirmed in Northern blot analysis (Figure 6A). Thus, we studied the role of MerA in protection under AGXX® stress in *S. aureus* COL using growth and survival phenotype assays. For phenotype analyses, we have chosen *S. aureus* COL as wild type, since all previous phenotype analysis of the *merA* mutant and its complemented strain under NaOCl stress were performed in *S. aureus* COL background (Loi et al., 2018). First, we noted that the growth of *S. aureus* COL is slightly impaired with 3 μg/ml AGXX® indicating that *S. aureus* COL is more sensitive to 3 μg/ml AGXX® compared to *S. aureus* USA300 (Figures 5A, 6B). The *merA* mutant showed a stronger growth inhibition with 3 μg/ml AGXX® than the wild type. Complementation of the *merA* mutant with plasmid-encoded *merA* restored the growth to wild type level (Figure 6B). The *merA* mutant was also more sensitive to killing by the lethal dose of 10 μg/ml AGXX® stress compared to the wild type (Figure 6C). The survival phenotype of the *merA* mutant could be restored back to wild type level after complementation with plasmid-encoded *merA*. This indicates that MerA provides protection against oxidative stress that is encountered during AGXX® treatment in *S. aureus*. MerA might help to reduce oxidized protein disulfides or LMW thiol disulfides, which remains to be elucidated. These phenotype results identified important roles of the LMW thiol BSH and the disulfide reductase MerA in the defense against strong disulfide stress encountered from ROS generation during AGXX® exposure in *S. aureus* USA300.

## Discussion

The novel antimicrobial coating AGXX® has a strong killing effect on a broad range of Gram-positive and Gram-negative bacteria and was shown to inhibit biofilm formation in the MRSA strain *S. aureus* 04-02981 and in a clinical *E. faecalis* isolate (Guridi et al., 2015; Clauss-Lendzian et al., 2018; Vaishampayan et al., 2018). Previous RNA-seq transcriptomics analysis in *S. aureus* revealed the downregulation of genes encoding virulence factors regulated by the Agr system (Vaishampayan et al., 2018). It was postulated that AGXX® inhibits the Agr system and thereby acts as potential biofilm inhibitor as main killing mode (Vaishampayan et al., 2018). In our RNA-seq experiments, we could not confirm the significant downregulation of the *agrBDCA* operon upon AGXX® stress and observed rather a 2–3-fold up-regulation of the α- and γ-hemolysins (*hla, hlgABC*) and significantly down-regulation of surface proteins *spa, sdrC, sdrD, sle1* in the transcriptome. Thus, our analysis suggests rather a slightly increased Agr activity under sub-lethal AGXX® stress in *S. aureus*. The Agr system is activated by autoinducer peptides that are induced by high cell density after the entry into the stationary phase (Novick, 2003; Queck et al., 2008). Since AGXX® exposure caused a growth defect, this could lead to activation of the Agr system in our study resulting in increased toxin and decreased surface protein expression. The discrepancies between the RNAseq results of the previous study (Vaishampayan et al., 2018) and our work might be related to the different AGXX® coating surfaces. We used small AGXX® microparticles (1.5–2.5 μM), which have a large volume/surface area, while larger AGXX® coated metal sheets (24 cm^2^) were used in the previous study (Vaishampayan et al., 2018). Thus, bacteria might be exposed more equally to the small microparticles in the shaking bacterial cultures, while metal sheets may not contact all bacteria in the shaking culture simultaneously.

However, previous analyses in *E. faecalis* revealed also the formation of ROS, such as H_2_O_2_ and hydroxyl radicals at the surface of AGXX® coatings as measured using spectroscopic methods (Clauss-Lendzian et al., 2018). These data point to a major role of ROS in the killing mode of AGXX® in bacteria. It is postulated that an electric field is formed between the transition metals silver and ruthenium leading to step-wise electron transfer to molecular oxygen and subsequent ROS formation (Guridi et al., 2015). Transcriptome in *E. faecalis* supported the oxidative stress mode of action of AGXX® since expression of genes for thioredoxins, catalase, superoxide dismutase and glutathione synthetase was highly upregulated (Clauss-Lendzian et al., 2018). In this study, we used a combination of RNA-seq transcriptomics, analyses for protein *S*-bacillthiolations and protein aggregations, redox biosensor measurements and mutant phenotype analyses to elucidate the thiol-reactive mode of action of AGXX® coating in *S. aureus* USA300.

The transcriptome results identified a striking overlap of the AGXX® response with the RNA-seq signature caused by the strong oxidant and microbicidal agent HOCl (Loi et al., 2018). Among the most strongly up-regulated oxidative stress specific regulons are the HypR, PerR, and QsrR regulons that are induced by H_2_O_2_, HOCl, and quinones in *S. aureus* (Horsburgh et al., 2001; Loi et al., 2018). The homologous HypR, PerR, and YodB regulons were also upregulated by HOCl stress in *Bacillus subtilis* (Chi et al., 2011). This confirms the generation of various forms of ROS, such as the less reactive H_2_O_2_, the strong oxidant hydroxyl radical and possibly quinones as the killing mode of AGXX® in *S. aureus* USA300. The formation of quinones is supported by the strong induction of the *mhqRDE* operon (Figures 2, 3). The *mhqRED* operon encodes for homologs of the quinone sensing MhqR repressor in *B. subtilis*, the phospholipase/carboxylesterase MhqE and the ring-cleavage dioxygenase MhqD, which specifically respond to quinones in *B. subtilis* (Töwe et al., 2007). Based on this transcriptome signature, we confirmed the generation of highly reactive ROS and quinones at the AGXX® coating in *S. aureus* as the main microbicidal mode of action.

In addition to ROS generation, previous studies suggested that free Ag^+^ ions could be released by AGXX® that might affect the metal homeostasis in bacteria (Guridi et al., 2015; Clauss-Lendzian et al., 2018). Transcriptome studies of the AGXX® response in *E. faecalis* showed the strong induction of the *copZA* operon, which also responds to elevated levels of Cu^+^ and Ag^+^ (Clauss-Lendzian et al., 2018). The activation of copper signaling and induction of the oxidative stress response by Ag^+^ was shown also in *E. coli* using a transcriptome study (McQuillan and Shaw, 2014). In *S. aureus*, the *copZA* operon is regulated by the Cu^+^ sensing CsoR repressor (Grossoehme et al., 2011) and is 48–56-fold induced by AGXX®. The *copZA* operon encodes a copper-binding chaperone CopZ and copper-translocating P-type ATPase CopA, that could be involved in transport and export of excess Ag^+^ to avoid silver intoxification. Moreover, we found strong induction of the CstR regulon which responds to hydrogen sulfide and nitroxyl (HNO) in *S. aureus* (Luebke et al., 2013, 2014; Peng et al., 2017). AGXX® might cause thiol-oxidation of the Cys residues of CstR resulting in inactivation of CstR and derepression of the *cstAB* operon encoding a thiosulfate sulfurtransferase and persulfide dioxygenase-sulfurtransferase (Higgins et al., 2015; Shen et al., 2015). Since CstR is a close homolog to CsoR (Grossoehme et al., 2011), inactivation of CsoR could similarly occur by thiol-oxidation of its Cu^+^-binding Cys residues leading to *copZA* derepression. In addition, our results revealed the strong induction of the Fur and Zur regulons under AGXX® that are indicative for iron and zinc starvation (Moore and Helmann, 2005; Chandrangsu et al., 2017). The Fur and Zur repressors harbor structural Zn-redox switch motifs that are susceptible for oxidation by the strong oxidant HOCl as shown in several redox proteomics studies in *E. coli* (Ilbert et al., 2006; Winter et al., 2008), *S. aureus* (Imber et al., 2018a) and *Mycobacterium smegmatis* (Hillion et al., 2017). Thus, oxidation of metal binding Cys ligands in Fur and Zur could trigger zinc release and the subsequent zinc and iron starvation responses.

Highly up-regulated under AGXX® stress are further the CtsR and HrcA-controlled chaperones and proteases of the protein quality control machinery involved in repair and degradation of oxidatively damaged and unfolded proteins (Frees et al., 2003, 2004, 2007, 2014). Among the Clp proteolytic and ATPase subunits, we identified *clpP, clpB* and the *mcsAB-clpC-ctsR* operon as ~50–150-fold up-regulated under AGXX® exposure. These results are in agreement with previous RNA-seq studies, showing the strong induction of *clpB, clpC* and *ctsR* genes in *S. aureus* and of *clpB* and *clpE* in *E. faecalis* (Clauss-Lendzian et al., 2018; Vaishampayan et al., 2018). The HrcA-dependent *groESL* operon was also highly induced in our and previous RNA-seq studies (Clauss-Lendzian et al., 2018; Vaishampayan et al., 2018), indicating that proteins might undergo thiol-oxidations and aggregation under AGXX® stress. Using a protein aggregation assay, we found higher amounts of aggregated proteins in the AGXX® treated proteome samples in agreement to the induction of the CtsR and HrcA regulons.

We have previously shown that HOCl causes >10% increased protein thiol-oxidation for 58 proteins in the redox proteome of *S. aureus* which includes five proteins that are modified by protein *S*-bacillithiolation (Chandrangsu et al., 2018; Imber et al., 2018a). The glycolytic GapDH was identified as the most abundant *S*-bacillithiolated protein under HOCl stress in the proteome. GapDH activity was inhibited by *S*-bacillithiolation to prevent its overoxidation to irreversible Cys sulfonic acid (Chandrangsu et al., 2018; Imber et al., 2018a). Here, we showed that protein *S*-bacillithiolation of GapDH is similar strongly increased under AGXX® treatment in *S. aureus* USA300. This further confirms the thiol-reactive mode of action of AGXX® in *S. aureus*. The increased formation of BSH mixed protein disulfides is accompanied by an oxidized shift in *E*_BSH_ which was measured using our Brx-roGFP2 biosensor (Loi et al., 2017). Brx-roGFP2 was previously shown to respond specifically to low levels of BSSB *in vitro* and was strongly oxidized by 50–100 μM HOCl in *S. aureus* cells *in vivo*. The Brx-roGFP2 biosensor was nearly fully oxidized after 60 min of sub-lethal 4 μg/ml AGXX® treatment in *S. aureus*. This clearly indicates that AGXX® affects the redox balance in *S. aureus*.

Since BSH is involved in protein *S*-bacillithiolation under AGXX® stress, we performed phenotype assays of the *bshA* mutant to investigate if BSH protects *S. aureus* from ROS encountered during AGXX® exposure. Our results revealed a strong growth and survival defect of the *bshA* mutant under sub-lethal and lethal doses of AGXX®. Thus, the LMW thiol BSH plays an important role in protection against microbicidal ROS produced by AGXX® exposure in *S. aureus*. Apart from BSH, the HypR-controlled disulfide reductase MerA (Loi et al., 2018) is involved in the defense against ROS produced by AGXX®. This supports the increased formation of protein disulfides and LMW thiol disulfides as potential substrates for MerA that remain to be elucidated (Loi et al., 2018).

Altogether, we have shown here that AGXX® causes a strong thiol-specific oxidative and metal stress response as well as strong protein damage as comprehensive mode of action in *S. aureus* USA300. The derepression of the Fur, Zur, CsoR and CstR regulons suggests that AGXX® impairs metal ion homeostasis by induction of Fe^2+^- and Zn^2+^-starvation responses as well as export systems for toxic Ag^+^ ions. The oxidative mode of action in the transcriptome was supported by increased protein *S*-bacillithiolation and protein aggregation as well as by an oxidative shift of the BSH redox potential as revealed using the Brx-roGFP2 biosensor. In addition, we showed that BSH and the disulfide reductase MerA play major roles in the defense against ROS produced under AGXX® stress.

Thus, the ROS-producing antimicrobial AGXX® has multiple modes of actions to kill pathogens that exclude the development of resistance mechanisms. The thiol-reactive AGXX® antimicrobial coating is also environmentally friendly, sustainable and can be applied on various medical devices, wound dressings, water pipelines and in the food industry and provides a promising strategy to efficiently combat multiple antibiotic resistant pathogens.

## Author Contributions

VL and HA conceived the project. VL, TB, and HA designed the experiments of the project. TB and JK contributed with RNA-seq transcriptomics analyses to the project. VL and TP performed the growth, survival, biosensor experiments and protein *S*-bacillithiolation assays. The AGXX® transcriptome treemap was constructed by JB. HA wrote the manuscript. All authors read and approved the manuscript.

### Conflict of Interest Statement

The authors declare that the research was conducted in the absence of any commercial or financial relationships that could be construed as a potential conflict of interest.

## Abbreviations

Ag: silver
AGXX®: antimicrobial coating of Ag^+^ and Ru^+^ ions
BSH: bacillithiol
BSSB: oxidized bacillithiol disulfide
Brx: bacilliredoxins
Brx-roGFP2: Brx fused to roGFP2
CFU: colony-forming unit
DTT: dithiothreitol
*E*_BSH_: bacillithiol redox potential
EDTA: ethylenediaminetetraacetic acid
GapDH: glyceraldehyde-3-phosphate dehydrogenase
H_2_O_2_: hydrogen peroxide
HOCl: hypochloric acid
LB: Luria Bertani
LMW thiol: low molecular weight thiol
MRSA: methicillin-resistant *Staphylococcus aureus*
NADPH: nicotinamide adenine dinucleotide phosphate
NaOCl: sodium hypochlorite
NEM: N-ethylmaleimide
NP-40: Nonidet P-40
OD_500_: optical density at 500 nm
OxD: oxidation degree of the Brx-roGFP2 biosensor
roGFP2: redox-sensitive GFP2
ROS: reactive oxygen species
Ru: ruthenium.

## References

[B1] Abomoelak B. Hoye E. A. Chi J. Marcus S. A. Laval F. Bannantine J. P.. (2009). *mosR*, a novel transcriptional regulator of hypoxia and virulence in *Mycobacterium tuberculosis*. J. Bacteriol. 191, 5941–5952. 10.1128/JB.00778-09 19648248 PMC2747884

[B2] Archer G. L. (1998). *Staphylococcus aureus*: a well-armed pathogen. Clin. Infect. Dis. 26, 1179–1181. 10.1086/520289 9597249

[B3] Beavers W. N. Skaar E. P. (2016). Neutrophil-generated oxidative stress and protein damage in *Staphylococcus aureus*. Pathog. Dis. 74:ftw060. 10.1093/femspd/ftw060 27354296 PMC5975594

[B4] Boucher H. W. Corey G. R. (2008). Epidemiology of methicillin-resistant *Staphylococcus aureus*. Clin. Infect. Dis. 46 (Suppl. 5), S344–S349. 10.1086/533590 18462089

[B5] Chandrangsu P. Loi V. V. Antelmann H. Helmann J. D. (2018). The role of bacillithiol in Gram-positive *Firmicutes*. Antioxid. Redox Signal. 28, 445–462. 10.1089/ars.2017.7057 28301954 PMC5790435

[B6] Chandrangsu P. Rensing C. Helmann J. D. (2017). Metal homeostasis and resistance in bacteria. Nat. Rev. Microbiol. 15, 338–350. 10.1038/nrmicro.2017.15 28344348 PMC5963929

[B7] Chen J.-Y. Wang L.-Y. Wu P.-W. (2010). Preparation and characterization of ruthenium films via an electroless deposition route. Thin Solid Films 518, 7245–7248. 10.1016/j.tsf.2010.04.086

[B8] Chen P. R. Bae T. Williams W. A. Duguid E. M. Rice P. A. Schneewind O.. (2006). An oxidation-sensing mechanism is used by the global regulator MgrA in *Staphylococcus aureus*. Nat. Chem. Biol. 2, 591–595. 10.1038/nchembio820 16980961

[B9] Chen P. R. Brugarolas P. He C. (2011). Redox signaling in human pathogens. Antioxid. Redox Signal. 14, 1107–1118. 10.1089/ars.2010.3374 20578795

[B10] Chen P. R. Nishida S. Poor C. B. Cheng A. Bae T. Kuechenmeister L.. (2009). A new oxidative sensing and regulation pathway mediated by the MgrA homologue SarZ in *Staphylococcus aureus*. Mol. Microbiol. 71, 198–211. 10.1111/j.1365-2958.2008.06518.x 19007410 PMC2698432

[B11] Chi B. K. Busche T. Van Laer K. Bäsell K. Becher D. Clermont L.. (2014). Protein *S*-mycothiolation functions as redox-switch and thiol protection mechanism in *Corynebacterium glutamicum* under hypochlorite stress. Antioxid. Redox Signal. 20, 589–605. 10.1089/ars.2013.5423 23886307 PMC3901351

[B12] Chi B. K. Gronau K. Mäder U. Hessling B. Becher D. Antelmann H. (2011). *S*-bacillithiolation protects against hypochlorite stress in *Bacillus subtilis* as revealed by transcriptomics and redox proteomics. Mol. Cell. Proteomics 10:M111009506. 10.1074/mcp.M111.009506 21749987 PMC3226405

[B13] Chi B. K. Roberts A. A. Huyen T. T. Bäsell K. Becher D. Albrecht D.. (2013). *S*-bacillithiolation protects conserved and essential proteins against hypochlorite stress in *Firmicutes* bacteria. Antioxid. Redox Signal. 18, 1273–1295. 10.1089/ars.2012.4686 22938038 PMC3584511

[B14] Clauss-Lendzian E. Vaishampayan A. De Jong A. Landau U. Meyer C. Kok J.. (2018). Stress response of a clinical *Enterococcus faecalis* isolate subjected to a novel antimicrobial surface coating. Microbiol. Res. 207, 53–64. 10.1016/j.micres.2017.11.006 29458868

[B15] Dooley C. T. Dore T. M. Hanson G. T. Jackson W. C. Remington S. J. Tsien R. Y. (2004). Imaging dynamic redox changes in mammalian cells with green fluorescent protein indicators. J. Biol. Chem. 279, 22284–22293. 10.1074/jbc.M312847200 14985369

[B16] Dubbs J. M. Mongkolsuk S. (2007). Peroxiredoxins in bacterial antioxidant defense. Subcell. Biochem. 44, 143–193. 10.1007/978-1-4020-6051-9_7 18084893

[B17] Falord M. Mäder U. Hiron A. Débarbouillé M. Msadek T. (2011). Investigation of the *Staphylococcus aureus* GraSR regulon reveals novel links to virulence, stress response and cell wall signal transduction pathways. PLoS ONE 6:e21323. 10.1371/journal.pone.0021323 21765893 PMC3128592

[B18] Foster T. J. (2004). The *Staphylococcus aureus* “superbug”. J. Clin. Invest. 114, 1693–1696. 10.1172/JCI200423825 15599392 PMC535074

[B19] Frees D. Chastanet A. Qazi S. Sørensen K. Hill P. Msadek T.. (2004). Clp ATPases are required for stress tolerance, intracellular replication and biofilm formation in *Staphylococcus aureus*. Mol. Microbiol. 54, 1445–1462. 10.1111/j.1365-2958.2004.04368.x 15554981

[B20] Frees D. Gerth U. Ingmer H. (2014). Clp chaperones and proteases are central in stress survival, virulence and antibiotic resistance of *Staphylococcus aureus*. Int. J. Med. Microbiol. 304, 142–149. 10.1016/j.ijmm.2013.11.009 24457183

[B21] Frees D. Qazi S. N. Hill P. J. Ingmer H. (2003). Alternative roles of ClpX and ClpP in *Staphylococcus aureus* stress tolerance and virulence. Mol. Microbiol. 48, 1565–1578. 10.1046/j.1365-2958.2003.03524.x 12791139

[B22] Frees D. Savijoki K. Varmanen P. Ingmer H. (2007). Clp ATPases and ClpP proteolytic complexes regulate vital biological processes in low GC, Gram-positive bacteria. Mol. Microbiol. 63, 1285–1295. 10.1111/j.1365-2958.2007.05598.x 17302811

[B23] Grass G. Rensing C. Solioz M. (2011). Metallic copper as an antimicrobial surface. Appl. Environ. Microbiol. 77, 1541–1547. 10.1128/AEM.02766-10 21193661 PMC3067274

[B24] Groitl B. Dahl J. U. Schroeder J. W. Jakob U. (2017). *Pseudomonas aeruginosa* defense systems against microbicidal oxidants. Mol. Microbiol. 106, 335–350. 10.1111/mmi.13768 28795780 PMC5653425

[B25] Grossoehme N. Kehl-Fie T. E. Ma Z. Adams K. W. Cowart D. M. Scott R. A.. (2011). Control of copper resistance and inorganic sulfur metabolism by paralogous regulators in *Staphylococcus aureus*. J. Biol. Chem. 286, 13522–13531. 10.1074/jbc.M111.220012 21339296 PMC3075698

[B26] Gupta A. Matsui K. Lo J. F. Silver S. (1999). Molecular basis for resistance to silver cations in *Salmonella*. Nat. Med. 5, 183–188. 10.1038/5545 9930866

[B27] Guridi A. Diederich A. K. Aguila-Arcos S. Garcia-Moreno M. Blasi R. Broszat M.. (2015). New antimicrobial contact catalyst killing antibiotic resistant clinical and waterborne pathogens. Mater. Sci. Eng. C Mater. Biol. Appl. 50, 1–11. 10.1016/j.msec.2015.01.080 25746238

[B28] Heiss A. Freisinger B. Held-Föhn E. (2017). Enhanced antibacterial activity of silver-ruthenium coated hollow microparticles. Biointerphases 12:05G608. 10.1116/1.5003803 29212331

[B29] Higgins K. A. Peng H. Luebke J. L. Chang F. M. Giedroc D. P. (2015). Conformational analysis and chemical reactivity of the multidomain sulfurtransferase, *Staphylococcus aureus* CstA. Biochemistry 54, 2385–2398. 10.1021/acs.biochem.5b00056 25793461

[B30] Highlander S. K. Hultén K. G. Qin X. Jiang H. Yerrapragada S. Mason E. O. Jr.. (2007). Subtle genetic changes enhance virulence of methicillin resistant and sensitive *Staphylococcus aureus*. BMC Microbiol. 7:99. 10.1186/1471-2180-7-99 17986343 PMC2222628

[B31] Hilker R. Stadermann K. B. Schwengers O. Anisiforov E. Jaenicke S. Weisshaar B.. (2016). ReadXplorer 2-detailed read mapping analysis and visualization from one single source. Bioinformatics 32, 3702–3708. 10.1093/bioinformatics/btw541 27540267 PMC5167064

[B32] Hillion M. Antelmann H. (2015). Thiol-based redox switches in prokaryotes. Biol. Chem. 396, 415–444. 10.1515/hsz-2015-0102 25720121 PMC4438307

[B33] Hillion M. Bernhardt J. Busche T. Rossius M. Maaß S. Becher D.. (2017). Monitoring global protein thiol-oxidation and protein *S*-mycothiolation in *Mycobacterium smegmatis* under hypochlorite stress. Sci. Rep. 7:1195. 10.1038/s41598-017-01179-4 28446771 PMC5430705

[B34] Horsburgh M. J. Clements M. O. Crossley H. Ingham E. Foster S. J. (2001). PerR controls oxidative stress resistance and iron storage proteins and is required for virulence in *Staphylococcus aureus*. Infect. Immun. 69, 3744–3754. 10.1128/IAI.69.6.3744-3754.2001 11349039 PMC98383

[B35] Ilbert M. Graf P. C. Jakob U. (2006). Zinc center as redox switch–new function for an old motif. Antioxid. Redox Signal. 8, 835–846. 10.1089/ars.2006.8.835 16771674

[B36] Imber M. Huyen N. T. T. Pietrzyk-Brzezinska A. J. Loi V. V. Hillion M. Bernhardt J.. (2018a). Protein S-Bacillithiolation functions in thiol protection and redox regulation of the glyceraldehyde-3-phosphate dehydrogenase Gap in *Staphylococcus aureus* under hypochlorite stress. Antioxid. Redox Signal. 28, 410–430. 10.1089/ars.2016.6897 27967218 PMC5791933

[B37] Imber M. Loi V. V. Reznikov S. Fritsch V. N. Pietrzyk-Brzezinska A. J. Prehn J.. (2018b). The aldehyde dehydrogenase AldA contributes to the hypochlorite defense and is redox-controlled by protein S-bacillithiolation in *Staphylococcus aureus*. Redox Biol. 15, 557–568. 10.1016/j.redox.2018.02.001 29433022 PMC5975064

[B38] Imber M. Pietrzyk-Brzezinska A. J. Antelmann H. (2018c). Redox regulation by reversible protein *S*-thiolation in Gram-positive bacteria. Redox Biol. 20, 130–145. 10.1016/j.redox.2018.08.017 30308476 PMC6178380

[B39] Ji C. J. Kim J. H. Won Y. B. Lee Y. E. Choi T. W. Ju S. Y.. (2015). *Staphylococcus aureus* PerR is a hypersensitive hydrogen peroxide sensor using iron-mediated histidine oxidation. J. Biol. Chem. 290, 20374–20386. 10.1074/jbc.M115.664961 26134568 PMC4536443

[B40] Langmead B. Salzberg S. L. (2012). Fast gapped-read alignment with Bowtie 2. Nat. Methods 9, 357–359. 10.1038/nmeth.1923 22388286 PMC3322381

[B41] Lansdown A. B. (2010). A pharmacological and toxicological profile of silver as an antimicrobial agent in medical devices. Adv. Pharmacol. Sci. 2010:910686. 21188244 10.1155/2010/910686 PMC3003978

[B42] Lee J. W. Helmann J. D. (2006). The PerR transcription factor senses H2O2 by metal-catalysed histidine oxidation. Nature 440, 363–367. 10.1038/nature04537 16541078

[B43] Li H. Handsaker B. Wysoker A. Fennell T. Ruan J. Homer N.. (2009). The sequence alignment/Map format and SAMtools. Bioinformatics 25, 2078–2079. 10.1093/bioinformatics/btp352 19505943 PMC2723002

[B44] Livermore D. M. (2000). Antibiotic resistance in staphylococci. Int. J. Antimicrob. Agents 16 (Suppl. 1), S3–10. 10.1016/S0924-8579(00)00299-5 11137402

[B45] Loi V. V. Busche T. Tedin K. Bernhardt J. Wollenhaupt J. Huyen N. T. T.. (2018). Redox-sensing under hypochlorite stress and infection conditions by the Rrf2-family repressor HypR in *Staphylococcus aureus*. Antioxid. Redox Signal. 29, 615–636. 10.1089/ars.2017.7354 29237286 PMC6067689

[B46] Loi V. V. Harms M. Müller M. Huyen N. T. T. Hamilton C. J. Hochgräfe F.. (2017). Real-time imaging of the bacillithiol redox potential in the human pathogen *Staphylococcus aureus* using a genetically encoded bacilliredoxin-fused redox biosensor. Antioxid. Redox Signal. 26, 835–848. 10.1089/ars.2016.6733 27462976 PMC5444506

[B47] Loi V. V. Rossius M. Antelmann H. (2015). Redox regulation by reversible protein *S*-thiolation in bacteria. Front. Microbiol. 6:187. 10.3389/fmicb.2015.00187 25852656 PMC4360819

[B48] Love M. I. Huber W. Anders S. (2014). Moderated estimation of fold change and dispersion for RNA-seq data with DESeq2. Genome Biol. 15:550. 10.1186/s13059-014-0550-8 25516281 PMC4302049

[B49] Lowy F. D. (1998). *Staphylococcus aureus* infections. N. Engl. J. Med. 339, 520–532. 10.1056/NEJM199808203390806 9709046

[B50] Luebke J. L. Arnold R. J. Giedroc D. P. (2013). Selenite and tellurite form mixed seleno- and tellurotrisulfides with CstR from *Staphylococcus aureus*. Metallomics 5, 335–342. 10.1039/c3mt20205d 23385876 PMC3714221

[B51] Luebke J. L. Shen J. Bruce K. E. Kehl-Fie T. E. Peng H. Skaar E. P.. (2014). The CsoR-like sulfurtransferase repressor (CstR) is a persulfide sensor in *Staphylococcus aureus*. Mol. Microbiol. 94, 1343–1360. 10.1111/mmi.12835 25318663 PMC4264537

[B52] Mäder U. Nicolas P. Depke M. Pané-Farré J. Debarbouille M. Van Der Kooi-Pol M. M.. (2016). *Staphylococcus aureus* transcriptome architecture: from laboratory to infection-mimicking conditions. PLoS Genet. 12:e1005962. 10.1371/journal.pgen.1005962 27035918 PMC4818034

[B53] Maillard J. Y. Hartemann P. (2013). Silver as an antimicrobial: facts and gaps in knowledge. Crit. Rev. Microbiol. 39, 373–383. 10.3109/1040841X.2012.713323 22928774

[B54] McQuillan J. S. Shaw A. M. (2014). Differential gene regulation in the Ag nanoparticle and Ag(+)-induced silver stress response in *Escherichia coli*: a full transcriptomic profile. Nanotoxicology 8 (Suppl. 1), 177–184. 10.3109/17435390.2013.870243 24392705

[B55] Mehlan H. Schüler F. Weiss S. Schuler J. Fuchs S. Riedel K.. (2013). Data visualization in environmental proteomics. Proteomics 13, 2805–2821. 10.1002/pmic.201300167 23913834

[B56] Moore C. M. Helmann J. D. (2005). Metal ion homeostasis in *Bacillus subtilis*. Curr. Opin. Microbiol. 8, 188–195. 10.1016/j.mib.2005.02.007 15802251

[B57] Newton G. L. Fahey R. C. Rawat M. (2012). Detoxification of toxins by bacillithiol in *Staphylococcus aureus*. Microbiology 158, 1117–1126. 10.1099/mic.0.055715-0 22262099 PMC3949421

[B58] Novick R. P. (2003). Autoinduction and signal transduction in the regulation of staphylococcal virulence. Mol. Microbiol. 48, 1429–1449. 10.1046/j.1365-2958.2003.03526.x 12791129

[B59] Padiadpu J. Baloni P. Anand K. Munshi M. Thakur C. Mohan A.. (2016). Identifying and tackling emergent vulnerability in drug-resistant *Mycobacteria*. ACS Infect Dis. 2, 592–607. 10.1021/acsinfecdis.6b00004 27759382

[B60] Pendleton J. N. Gorman S. P. Gilmore B. F. (2013). Clinical relevance of the ESKAPE pathogens. Expert Rev. Anti Infect. Ther. 11, 297–308. 10.1586/eri.13.12 23458769

[B61] Peng H. Shen J. Edmonds K. A. Luebke J. L. Hickey A. K. Palmer L. D.. (2017). Sulfide homeostasis and nitroxyl intersect via formation of reactive sulfur species in *Staphylococcus aureus*. mSphere 2, e00082–17. 10.1128/mSphere.00082-17 28656172 PMC5480029

[B62] Pförtner H. Burian M. S. Michalik S. Depke M. Hildebrandt P. Dhople V. M.. (2014). Activation of the alternative sigma factor SigB of *Staphylococcus aureus* following internalization by epithelial cells - an *in vivo* proteomics perspective. Int. J. Med. Microbiol. 304, 177–187. 10.1016/j.ijmm.2013.11.014 24480029

[B63] Pinochet-Barros A. Helmann J. D. (2018). Redox sensing by Fe^2+^ in bacterial fur family metalloregulators. Antioxid. Redox Signal. 29, 1858–1871. 10.1089/ars.2017.7359 28938859 PMC6217742

[B64] Poor C. B. Chen P. R. Duguid E. Rice P. A. He C. (2009). Crystal structures of the reduced, sulfenic acid, and mixed disulfide forms of SarZ, a redox active global regulator in *Staphylococcus aureus*. J. Biol. Chem. 284, 23517–23524. 10.1074/jbc.M109.015826 19586910 PMC2749125

[B65] Posada A. C. Kolar S. L. Dusi R. G. Francois P. Roberts A. A. Hamilton C. J.. (2014). Importance of bacillithiol in the oxidative stress response of *Staphylococcus aureus*. Infect. Immun. 82, 316–332. 10.1128/IAI.01074-13 24166956 PMC3911838

[B66] Pöther D. C. Gierok P. Harms M. Mostertz J. Hochgräfe F. Antelmann H.. (2013). Distribution and infection-related functions of bacillithiol in *Staphylococcus aureus*. Int. J. Med. Microbiol. 303, 114–123. 10.1016/j.ijmm.2013.01.003 23517692

[B67] Queck S. Y. Jameson-Lee M. Villaruz A. E. Bach T. H. Khan B. A. Sturdevant D. E.. (2008). RNAIII-independent target gene control by the *agr* quorum-sensing system: insight into the evolution of virulence regulation in *Staphylococcus aureus*. Mol. Cell 32, 150–158. 10.1016/j.molcel.2008.08.005 18851841 PMC2575650

[B68] Shen J. Keithly M. E. Armstrong R. N. Higgins K. A. Edmonds K. A. Giedroc D. P. (2015). *Staphylococcus aureus* CstB is a novel multidomain persulfide dioxygenase-sulfurtransferase involved in hydrogen sulfide detoxification. Biochemistry 54, 4542–4554. 10.1021/acs.biochem.5b00584 26177047 PMC4874178

[B69] Sun F. Ding Y. Ji Q. Liang Z. Deng X. Wong C. C.. (2012). Protein cysteine phosphorylation of SarA/MgrA family transcriptional regulators mediates bacterial virulence and antibiotic resistance. Proc. Natl. Acad. Sci. U.S.A. 109, 15461–15466. 10.1073/pnas.1205952109 22927394 PMC3458358

[B70] Tam Le T. Eymann C. Albrecht D. Sietmann R. Schauer F. Hecker M.. (2006). Differential gene expression in response to phenol and catechol reveals different metabolic activities for the degradation of aromatic compounds in *Bacillus subtilis*. Environ. Microbiol. 8, 1408–1427. 10.1111/j.1462-2920.2006.01034.x 16872404

[B71] Tomoyasu T. Arsene F. Ogura T. Bukau B. (2001). The C terminus of sigma(32) is not essential for degradation by FtsH. J. Bacteriol. 183, 5911–5917. 10.1128/JB.183.20.5911-5917.2001 11566990 PMC99669

[B72] Töwe S. Leelakriangsak M. Kobayashi K. Van Duy N. Hecker M. Zuber P.. (2007). The MarR-type repressor MhqR (YkvE) regulates multiple dioxygenases/glyoxalases and an azoreductase which confer resistance to 2-methylhydroquinone and catechol in *Bacillus subtilis*. Mol. Microbiol. 66, 40–54. 10.1111/j.1365-2958.2007.05891.x 17725564

[B73] Tung Q. N. Linzner N. Loi V. V. Antelmann H. (2018). Application of genetically encoded redox biosensors to measure dynamic changes in the glutathione, bacillithiol and mycothiol redox potentials in pathogenic bacteria. Free Radic. Biol. Med. 128, 84–96. 10.1016/j.freeradbiomed.2018.02.018 29454879

[B74] Vaishampayan A. De Jong A. Wight D. J. Kok J. Grohmann E. (2018). A novel antimicrobial coating represses biofilm and virulence-related genes in methicillin-resistant *Staphylococcus aureus*. Front. Microbiol. 9:221. 10.3389/fmicb.2018.00221 29497410 PMC5818464

[B75] Villapún V. M. Dover L. G. Cross A. González S. (2016). Antibacterial metallic touch surfaces. Materials 9:736. 10.3390/ma9090736 28773856 PMC5457048

[B76] Wetzstein M. Völker U. Dedio J. Löbau S. Zuber U. Schiesswohl M.. (1992). Cloning, sequencing, and molecular analysis of the *dnaK* locus from *Bacillus subtilis*. J. Bacteriol. 174, 3300–3310. 10.1128/jb.174.10.3300-3310.1992 1339421 PMC205999

[B77] Winter J. Ilbert M. Graf P. C. Ozcelik D. Jakob U. (2008). Bleach activates a redox-regulated chaperone by oxidative protein unfolding. Cell 135, 691–701. 10.1016/j.cell.2008.09.024 19013278 PMC2606091

[B78] Winterbourn C. C. Kettle A. J. (2013). Redox reactions and microbial killing in the neutrophil phagosome. Antioxid. Redox Signal. 18, 642–660. 10.1089/ars.2012.4827 22881869

[B79] Winterbourn C. C. Kettle A. J. Hampton M. B. (2016). Reactive oxygen species and neutrophil function. Annu. Rev. Biochem. 85, 765–792. 10.1146/annurev-biochem-060815-014442 27050287

